# De Hass-van Alphen and magnetoresistance reveal predominantly single-band transport behavior in PdTe_2_

**DOI:** 10.1038/srep31554

**Published:** 2016-08-12

**Authors:** Yongjian Wang, Jinglei Zhang, Wenka Zhu, Youming Zou, Chuanying Xi, Long Ma, Tao Han, Jun Yang, Jingrong Wang, Junmin Xu, Lei Zhang, Li Pi, Changjin Zhang, Yuheng Zhang

**Affiliations:** 1 High Magnetic Field Laboratory, Chinese Academy of Sciences and University of Science and Technology of China, Hefei 230026, China; 2 Collaborative Innovation Center of Advanced Microstructures, Nanjing University, Nanjing, 210093, China

## Abstract

Research on two-dimensional transition metal dichalcogenides (TMDs) has grown rapidly over the past several years, from fundamental studies to the development of next generation technologies. Recently, it has been reported that the *MX*_2_-type PdTe_2_ exhibits superconductivity with topological surface state, making this compound a promising candidate for investigating possible topological superconductivity. However, due to the multi-band feature of most of TMDs, the investigating of magnetoresistance and quantum oscillations of these TMDs proves to be quite complicated. Here we report a combined de Hass-van Alphen effect and magnetoresistance studies on the PdTe_2_ single crystal. Our high-field de Hass-van Alphen data measured at different temperature and different tilting angle suggest that though these is a well-defined multi-band feature, a predominant oscillation frequency has the largest oscillation magnitude in the fast Fourier transformation spectra, which is at least one order of magnitude larger than other oscillation frequencies. Thus it is likely that the transport behavior in PdTe_2_ system can be simplified into a single-band model. Meanwhile, the magnetoresistance results of the PdTe_2_ sample can be well-fitted according to the single-band models. The present results could be important in further investigation of the transport behaviors of two-dimensional TMDs.

The transition metal dichalcogenides (TMD) have recently become the focus of fundamental research and technological applications due to their unique crystal structures, a wide range of chemical compositions, novel and intriguing properties with potential applications in field effect transistors, optoelectronic devices, topological insulators, electrocatalysts, and so on^1^. Among them, the *MX*_2_-type transition metal dichalcogenides, such as TaSe_2_, TaS_2_, IrTe_2_, WTe_2_, MoS_2_, and MoSe_2_, have attracted great attention due to their rich physical properties like charge-density wave^2^,^3^,^4^, Mott-insulator to metal transition^4^, superconductivity^5^,^6^,^7^, catalysis of chemical reactions^8^, extremely large magneto-resistance^9^, and potential technological applications^9^,^10^,^11^. A thorough investigation of the electronic structures of these compounds is important for understanding their physical properties and exploring for new phenomena.

Recently, it has been discovered that the *MX*_2_-type PdTe_2_ and its Cu-intercalated counterpart Cu_*x*_PdTe_2_ exhibit superconductivity with transition temperature (*T*_*c*_) of about 1.7 K^12^. The following angle-resolved photoemission spectroscpoy results and theoretical calculations have revealed the existence of topologically nontrivial surface state with Dirac cone in PdTe_2_^13^, which offers a new material base for understanding the superconducting mechanism in the transition metal dichalcogenide compounds. More importantly, the coexistence of topological surface state with bulk superconductivity may offer an important material candidate for investigating possible topological superconductivity^14^,^15^, which is of particular importance both in fundamental physics such as the searching of long-sought yet elusive Majorana Fermions and in practical applications in the next-generation spintronics technologies.

The electronic structure of the PdTe_2_ compound has been investigated both theoretically and experimentally^16^,^17^,^18^,^19^. It turns out that the PdTe_2_ exhibits a complex Fermi surface topology with a three-fold symmetry. A small electron-like spot appears around the Γ point, which is surrounded by six small electron-like spots locating near the midpoint between Γ and *K*. The complicated multiband Fermi surface makes it rather difficult in the understanding of the transport properties and possible topological characters. In this paper, we report the de Haas-van Alphen effect (dHvA effect) and magnetoresistance (MR)results of the PdTe_2_ single crystal. The dHvA results clearly suggest that only one kind of carrier with certain relaxation rate dominates the electronic structure of the PdTe_2_ compound, which makes the situation more simple. The dominance of one kind of carrier is further supported by the MR results, where the Kohler’s law is well obeyed. The present observation could be important in further investigation of the electronic structure and transport behaviors of the PdTe_2_-related compound.

Figure 1 gives the powder x-ray diffraction pattern and the single crystal x-ray diffraction pattern of the PdTe_2_ sample. The powder x-ray diffraction pattern suggests that the PdTe_2_ sample is well crystallized in the CdI_2_-type structure with the 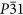 (No. 164) space group. The refined lattice parameters are *a* = *b* = 4.112 Å and *c* = 5.233 Å, which are consistent with previous reports^20^. The as-grown samples exhibit shining silvery surface with typical dimensions of 4 × 4 × 0.5 mm^3^. A picture of the cleaved single crystal is shown in the upper right corner of Fig. 1. The single crystal x-ray diffraction pattern is performed on the cleaved shining surface of the as-grown PdTe_2_ single crystal. The observed peaks can be indexed into (0 0 *n*) (with *n* being integers), indicating that the naturally cleaved surface is the basal *ab* plane. In the inset of Fig. 1 we show the temperature dependence of in-plane resistivity of the PdTe_2_ sample. A metallic behavior is observed at all temperature range. Below *T*_*c*_ ~ 2 K, the superconducting transition occurs. These results suggest that the obtained sample is high-quality PdTe_2_ single crystal sample.

Figure 2(a) shows the quantum oscillation data of the PdTe_2_ sample using the de Haas-van Alphen effect (dHvA effect) at *T* = 0.36 K. The inmagnetization quantum oscillations are probed via highly sensitive torque magnetometry methods which measure the magnetic susceptibility anisotropy of the sample. The experimental setup is schematically depicted in the inset of Fig. 2(a). The sample is mounted on the sample holder with the basal *ab* plane parallel to the top surface of the sample holder. The magnetic field is applied to the crystal with a tilt angle relative to the crystalline *c* axis. We find that at *θ* = 60°, the dHvA effect has the strongest oscillation amplitude comparing to other angles (the angel dependence of dHvA effect will be discussed later). The polynomial background has been subtracted and the dHvA effect exhibits good oscillation feature. The oscillatory pattern in *δC*/*C*_0_ is periodic in 1/*μ*_0_*H* and the oscillation arise from the successive empty of Landau levels when the field is increased (Fig. 2(b)). In the inset of Fig. 2(b) we plot the fast Fourier transformation (FFT) spectra of the oscillatory torque after subtracting the polynomial background. One can clearly see three peaks in the FFT spectra, which are labeled as F_1_, F_2_, and F_3_, respectively. We carefully check the position of these peaks and find that F_1_ = 121.5 T, F_2_ = 239 T, F_3_ = 283.2 T. Since F_2_ ≈ 2F_1_, the F_2_ can be considered as the multiple frequency signal of F_1_. Besides F_1_, there is a weak peak locating at F_3_ = 283.2 T, which is consistent with the multi-band feature of the PdTe_2_ compound^16^,^17^,^18^,^19^. However, the FFT peaks of F_3_ as well as other possible peaks derived from the multi-band feature of PdTe_2_ compound are all at least one order of magnitude less than the F_1_ peak. The dominant oscillation frequency is F_1_ = 121.5 T, which is in agreement with the early dHvA results by A. E. Dunsworth^16^. According to the Onsager relation 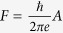, we can get the cross-sectional area of the Fermi surface normal to the field, that is *A* = 1.16 × 10^−2^ Å^−2^. The corresponding Fermi momentum is estimated to be *k*_*F*_ = 0.061 Å^−1^.

In order to obtain the effective mass and Dingle temperature of the charge carriers, we perform the dHvA measurements at various temperatures. The results are shown in Fig. 3(a), where the magnetic torque is plotted as a function of 1/*μ*_0_*H*. The dHvA oscillation can be described by the Lifshitz-Kosevich formula^21^,^22^,^23^,


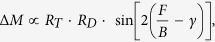


where *R*_*T*_ and *R*_*D*_ are the thermal damping factor and the Dingle damping factor, respectively, *γ* is a phase shift. The thermal damping factor is *R*_*T*_ = *X*/sin h(*X*), where 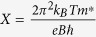. And the Dingle damping factor is 
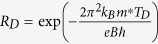
, where m* is the effective mass and *T*_*D*_ is the Dingle temperature. At a fixed magnetic field, the amplitude of the the quantum oscillation is given by *A* ∝ *R*_*T*_ · *R*_*D*_. Figure 3(b) shows the amplitude versus temperature at the fixed magnetic field of *H* = 12.4 T. We use the thermal damping factor *R*_*T*_ to fit these experimental data points and yields the cyclotron effective mass m* ≈ 0.13 *m*_*e*_. Based on the effective mass m* and the Fermi momentum *k*_*F*_, we obtain the related Fermi velocity *ν*_*F*_ ≈ 5.6 × 10^5^ m/s by using the formula *ν*_*F*_ = 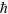 *k*_*F*_/*m*.

Dingle temperature *T*_*D*_ is related to the cyclotron relaxation rate with the formula 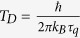
24. We get the Dingle temperature by fitting the Dingle plot at different magnetic fields. The experimental data and the fitting line are shown in the inset of Fig. 3(b). We find that the fitting line can well-reproduce the experimental data. The fit yields the Dingle temperature *T*_*D*_ = 30.4 ± 1.4 K. The cyclotron relaxation rate is estimated to be *τ*_*q*_ = 4 × 10^−14^ s.

From above de Hass-van Alphen oscillation data we notice that a predominant oscillatory frequency which is at least one order of magnitude larger than other oscillation frequencies. In order to clarify which band is corresponded to this oscillatory, we perform the dHvA effect experiments at different tilting angle relative to the crystalline *c* axis at the fixed temperature *T* = 1.5 K and compare the results with previous theoretical calculations and experimental data. The experimental setup is the same as that depicted in the inset of Fig. 2(a). It is found that the quantum oscillation has the strongest magnitude at around 60°. The magnitude of dHvA oscillation gradually decreases when the tilting angle is go away from 60°. Particularly, when the tilting angle is less than 16°, the oscillation signal is comparable with the noise signal. We make the fast Fourier transformation on each curve and get the dominant oscillation frequencies for every angle, which are given as the blue circles in Fig. 4(b). We use the two-dimensional (2*D*) Fermi surface model and three-dimensional (3*D*) ellipsoid Fermi surface model to fit the experimental data and We find that both the 2*D* Fermi surface and the 3*D* ellipsoid Fermi surface cannot well-reproduce the experimental data. The angle dependent dHvA results suggest a complicated Fermi surface topology of the PdTe_2_ compound, which is consistent with previous de Hass-van Alphen results^16^. We carefully compare the angle-dependent oscillatory frequency presented in Fig. 4(b) with previous band structure calculations and experimental band structure studies^16^,^17^. We find that both the high frequency branches and the medium frequency branches are not detected in the present de Hass-van Alphen results. At low frequency region, there are five branches: *ε*_1_, *ε*_2_, *ε*_3_, *ζ*, and *o*^16^. The branch *ζ* is a long ellipsoid or cylinder lying along *c*. The branches *ε*_1_, *ε*_2_, and *ε*_3_ are related and can be described by six long ellipsoids or cylinders which are 60° apart from each other and 40° tilted from *c*. And the branch *o* could be another orbit around the *ε*’s or a new piece of Fermi surface, which lies slightly above *ε*_1_. We compare the angle dependence of dHvA frequencies and the effective masses of our experimental data with those of the *ε*_1_, *ε*_2_, *ε*_3_, *ζ*, and *o* branches^16^,^17^. It is found that though the *ζ* branch has the same effective mass with the present data, its angle dependence of dHvA frequency is significant larger than the data given in Fig. 4(b). On the other hand, both the angle dependence of dHvA frequencies and the effective masses in our dHvA oscillations are comparable with those of the *ε* branches. Since the branches *ε*_1_, *ε*_2_, and *ε*_3_ are related with each other and are described by six long ellipsoids or cylinders which are 60° apart from each other, these branches cannot be distinguished in dHvA measurement. Thus it can be concluded that the predominant oscillatory frequency in the present de Hass-van Alphen data is related to the *ε* branches. This could probably explain the fact that the de Hass-van Alphen oscillations have the strongest magnitude at around 60°. That is, at 60°, each ellipsoid/cylinder contributes equally to the dHvA oscillations and the sum of the dHvA oscillations has the largest magnitude.

The present de Hass-van Alphen oscillation results suggest that the electronic structure in PdTe_2_ system could be simplified in a single-band model. In order to further investigate the magnetotransport behaviors of the PdTe_2_ compound, we perform the magnetoresistance measurements on the PdTe_2_ single crystal sample at different temperature. The results are shown in Fig. 5(a). At all temperatures, it is found that the resistivity increases with increasing the applied magnetic field, suggesting a positive magnetoresistance. The magnetoresistance decreases with increasing temperature. It is known that the Kohlers Rule is a suitable criterion for judging whether the transport properties of a given material is dominated by a single band or multi-band feature. According to the Kohlers Rule^25^, for a given metal with only one relaxation rate *τ*, the increase in resistivity in a magnetic field *H* relative to the zero-field value *ρ*_0_ is a universal function of *H*/*ρ*_0_, Δ*ρ*/*ρ*_0_ = *f(H*/*ρ*_0_), at all temperatures *T* and fields *H*. Here *f(H*/*ρ*_0_) is a universal function for a certain material, independent of temperature or impurity content. Note that this rule is only applicable to single-band metals. The Kohler plots for the PdTe_2_ single crystal sample are shown in Fig. 5(b) for temperatures between 0.36 K to 70 K and magnetic fields from 0 to 30 T. It is obvious that the magnetoresistance properties of the PdTe_2_ sample follow the Kohlers law well at all temperature. These facts suggest that despite of the multiband character in PdTe_2_, only one kind of charge carrier dominates the transport behavior.

We also find that the magnetoresistance of PdTe_2_ obeys the Δ*ρ*/*ρ*_0_ ∝ *H*^2^ criterion at low magnetic field (*H* ≤ 3 T), which is another evidence of the predominantly single-band transport behavior in this compound (see the inset in Fig. 5(b)). Interestingly, the magnetoresistance Δ*ρ*/*ρ*_0_ is approximately linear dependent on *H*^2^ under high magnetic field. The magnetoresistance does not saturate even when the applied magnetic field is as high as 30 Tesla. The Δ*ρ*/*ρ*_0_ value at 30 T is about 900% at 0.36 K. The large unsaturated magnetoresistance phenomenon is recently found in many topologically-related compounds^9^,^26^,^27^, probably meaning a common origin of the unsaturated magnetoresistance in these systems. This result seems to be consistent with the observation of topological surface state by recent angle-resolved photoemission spectroscopy experiments^13^.

The above de Hass-van Alphen and magnetoresistance results both point to a predominantly single-band feature in the PdTe_2_ compound. However, due to the lack of Shubnikov-de Hass results on the PdTe_2_ sample, we are not able to accurately determine which band dominates the transport properties of the present compound. In order to thoroughly understand the electronic structure of the PdTe_2_ compound, a systematic investigation on the magnetotransport properties is needed.

The identification of the topological superconducting state has become a big challenge in condensed matter physics and materials communities. The superconductivity induced by atomic intercalation in pristine topological insulator Bi_2_Se_3_ has attracted great attention in the investigation of possible topological superconductivity^14^,^15^,^28^,^29^. However, these intercalated materials usually suffer from serious chemical inhomogeneity. And there is no consensus on whether or not these materials are topological superconductors. The PdTe_2_, on the other hand, is much simple in chemical composition and lattice structure, making this compound a promising candidate for investigating possible topological superconductivity. The existence of topological surface state has been observed in PdTe_2_ by a recent angle-resolved photoemission spectroscopy study^13^. The next step would be the study of its superconducitng pairing symmetry and the searching of the Majorona Fermions at the vortex cores of the PdTe_2_ compound. Combining the simplicity in chemical composition and lattice structure, as well as the predominately single-band electronic structure, the PdTe_2_ compound could be served as an ideal material base in further investigation of the possible topological superconductivity.

In conclusion, by using de Hass-van Alphen effects and megnetoresistance we study the electronic structure and transport behavior of the two-dimensional TMD material PdTe_2_. Though the multi-band feature is well constructed in this material, we find the existence of a predominant oscillation frequency in the fast Fourier transformation spectra. Thus it is likely that a predominant single band electronic structure dominates the transport behavior of the PdTe_2_ compound, with other bands contribute very little. This result will be helpful in understanding the topological superconducting properties of this material, and also helps to realize the transport properties of related two-dimensional TMDs.

## Methods

Single crystal of PdTe_2_ was grown by melting stoichiometric mixtures of high-purity elements Pd (99.999%) and Te (99.997%) in a sealed evacuated quartz tube at 780 °C for 48 h, followed by a slow cooling to 500 °C at a rate of 3 °C/h. After that, the crystals were cooled with furnace to room temperature. The crystals can be easily cleaved into sheets with shiny mirror-like surfaces. The structure was checked by the Rigaku- TTR3 x-ray diffractometer using high-intensity graphite monochromatized Cu K_*α*_ radiation at room temperature. The orientation of *c* axis was perpendicular to the shining surface of the as-cleaved single crystal samples, which was confirmed by the single crystal x-ray diffraction measurements on the Rigaku- TTR3 x-ray diffractometer. The *b*-axis was determined by a back-reflection Laue x-ray photograph measurement.

The magnetoresistance measurements were performed using the standard four-probe technique. Quantum oscillations inmagnetization were probed via highly sensitive torque magnetometry methods which measure the magnetic susceptibility anisotropy of sample. With the tilted magnetic field *H* confined to the *x*-*z* plane and *M* in the same plane, the torque is 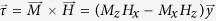. In paramagnetic state, it can be written as *τ* = *χ*_*z*_*H*_*z*_*H*_*x*_ − *χ*_*x*_*H*_*x*_*H*_*z*_ = Δ*χH*^2^sin *θ *cos *θ*, where Δ*χ* = *χ*_*z*_ − *χ*_*x*_ is the magnetic susceptibility anisotropy and *θ* is the tilt angle of *H* away from *z*. Throughout this paper, *θ* is defined as the angle between the magnetic field direction and the crystallographic *c* axis. Both magnetoresistance and torque measurements were performed on the Cell5 Water-Cooling Magnet of High Magnetic Field Laboratory of Chinese Academy of Sciences.

## Additional Information

**How to cite this article**: Wang, Y. *et al*. De Hass-van Alphen and magnetoresistance reveal predominantly single-band transport behavior in PdTe_2_. *Sci. Rep.* **6**, 31554; doi: 10.1038/srep31554 (2016).

## Figures and Tables

**Figure 1:**
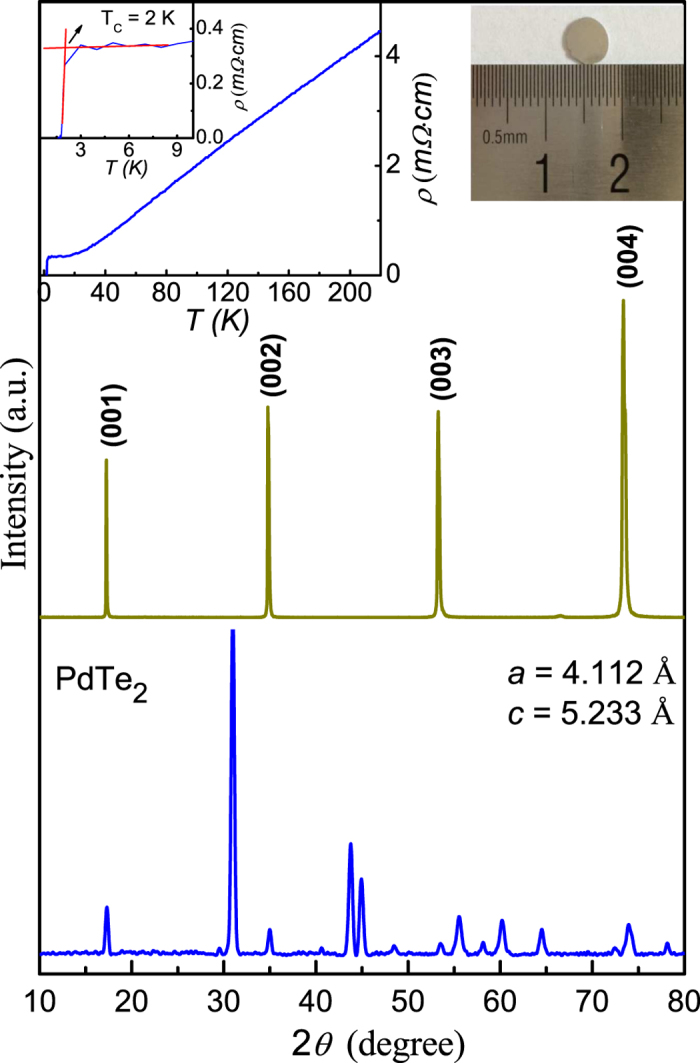
The powder and single-crystal x-ray diffraction pattern of the PdTe_2_ sample. The inset shows the temperature dependence of in-plane resistivity of the sample. The superconducting transition occurs with *T*_*c*_ = 2.0 K.

**Figure 2:**
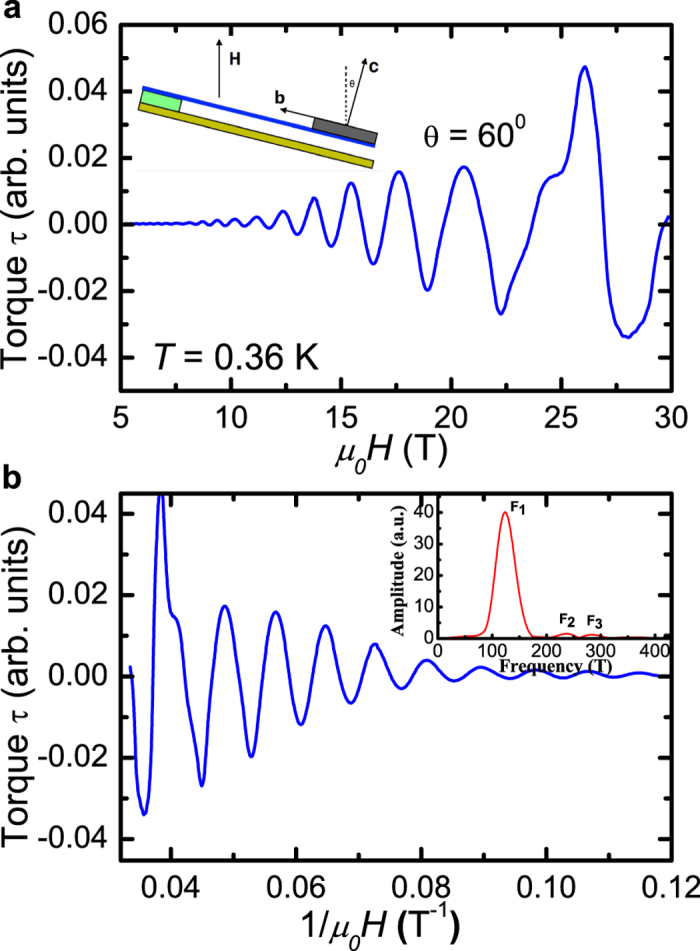
(**a**) The de Hass-van Alphen oscillations in the torque data with subtracting the polynomial background at 0.36 K. A schematic of the experimental setup is shown in upper left corner, where the magnetic field is applied to the crystal with a tilt angle relative to the crystalline *c* axis. (**b**) The de Hass-van Alphen oscillations ploted against 1/*μ*_0_*H*. The inset is a fast Fourier transformation (FFT) of the oscillatory torque after subtracting the polynomial background.

**Figure 3:**
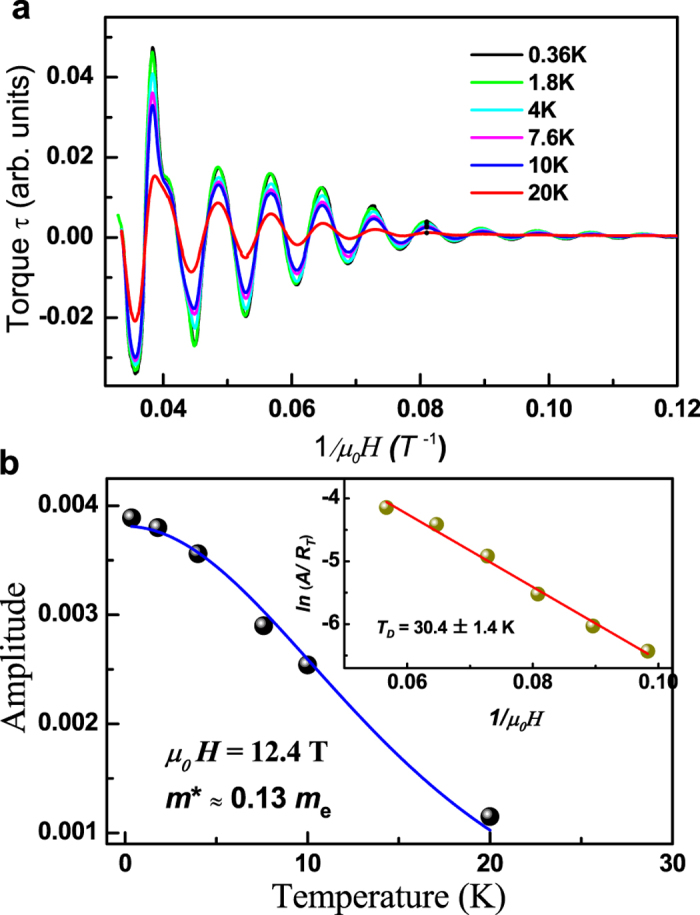
(**a**) The de Hass-van Alphen oscillations ploted against 1/*μ*_0_*H* at various temperatures. (**b**) The temperature dependence of the thermal damping factor of dHvA oscillation. The blue curve is a fit to the Lifshitz-Kosevich formula, from which we can extract the cyclotron effective mass m* ≈ 0.13 *m*_0_ and Fermi velocity *ν*_*F*_ ≈ 5.6 × 10^−5^ *m*/*s*. The inset of (**b**) shows the Dingle plot at different magnetic fields. The red line is a fit using the Lifshitz-Kosevich formula, which gives *T*_*D*_ = 30.4 ± 1.4 K.

**Figure 4:**
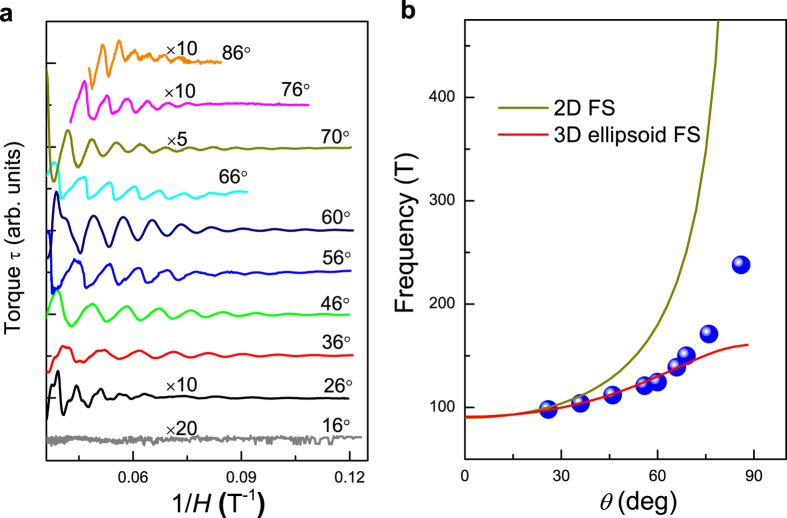
(**a**) The de Hass-van Alphen oscillations measured under different tilt angles. (**b**) The angle dependence of the dominant oscillation frequency at the temperature of 1.5 K. The solid lines are the fits to the Fermi surfaces using a two-dimensional Fermi surface and a three-dimensional ellipsoid Fermi surface model, respectively.

**Figure 5:**
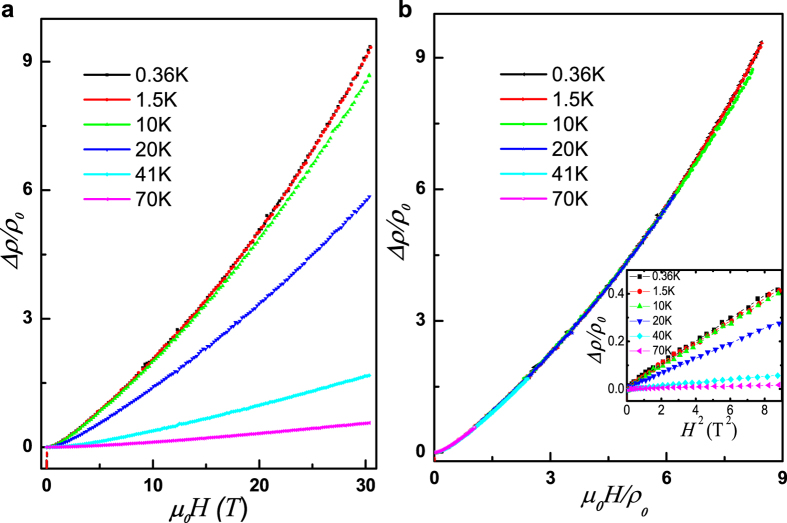
(**a**) The magnetic field dependence of in-plane resistance of the PdTe_2_ sample at different temperatures. (**b**) Kohler plot for the PdTe_2_ sample at different temperatures. It can be found that the Kohlers rule is obeyed at all temperatures. The inset plots the variation of Δ*ρ*/*ρ*_0_ against *H*^2^, where the Δ*ρ*/*ρ*_0_ ∝ *H*^2^ criterion is also obeyed when the magnetic field is less than 3 T.

## References

[b1] Wang H., Yuan H., Hong S. S. & Cui Y. Physical and chemical tuning of two-dimensional transition metal dichalcogenides. Chem. Soc. Rev. 44, 2664 (2015). 25474482 10.1039/c4cs00287c

[b2] Hajiyev P., Cong C. X., Qiu C. Y. & Yu T. Contrast and Raman spectroscopy study of single- and few-layered charge density wave material: 2H-TaSe_2_. Sci. Rep. 3, 2593 (2013). 24005335 10.1038/srep02593 PMC3763362

[b3] Tsen A. W. . Structure and control of charge density waves in two-dimensional 1T-TaS_2_. Proc. Natl. Acad. Sci. USA 112, 15054 (2015). 26598707 10.1073/pnas.1512092112 PMC4679066

[b4] Yu Y. J. . Gate-tunable phase transitions in thin flakes of 1T-TaS_2_. Nature Nanotech. 10, 270 (2015). 10.1038/nnano.2014.323 25622230

[b5] Sipos B. . From Mott state to superconductivity in 1T-TaS_2_. Nature Mater. 7, 960 (2008). 18997775 10.1038/nmat2318

[b6] Yang J. J. . Charge-orbital density wave and superconductivity in the strong spin-orbit coupled IrTe_2_:Pd. Phys. Rev. Lett. 108, 116402 (2012). 22540494 10.1103/PhysRevLett.108.116402

[b7] Zhang R. Y. . Superconductivity in potassium-doped metallic polymorphs of MoS_2_. Nano Lett. 16, 629 (2016). 26612060 10.1021/acs.nanolett.5b04361

[b8] Li Y. G. . MoS_2_ nanoparticles grown on graphene: An advanced catalyst for the hydrogen evolution reaction. J. Am. Chem. Soc. 133, 7296 (2011). 21510646 10.1021/ja201269b

[b9] Ali M. N. . Large, non-saturating magnetoresistance in WTe_2_. Nature 514, 205 (2014). 25219849 10.1038/nature13763

[b10] Yeh P. C. . Layer-dependent electronic structure of an atomically heavy two-dimensional dichalcogenide. Phys. Rev. B 91, 041407(R) (2015).

[b11] Chang T. R. . Prediction of an arc-tunable Weyl Fermion metallic state in Mo_*x*_W_1−*x*_Te_2_. Nature Commun. 7, 10639 (2016). 26875819 10.1038/ncomms10639 PMC4756349

[b12] Ryu G. Superconductivity in Cu-Intercalated CdI_2_-Type PdTe_2_. J. Supercond. Novel Magn. 28, 3275 (2015).

[b13] Liu Y. . Identification of topological surface state in PdTe_2_ superconductor by angle-resolved photoemission spectroscopy. Chin. Phys. Lett. 32, 067303 (2015).

[b14] Hor Y. S. . Superconductivity in Cu_*x*_Bi_2_Se_3_ and its Implications for Pairing in the Undoped Topological Insulator. Phys. Rev. Lett. 104, 057001 (2010). 20366785 10.1103/PhysRevLett.104.057001

[b15] Liu Z. H. . Superconductivity with topological surface state in Sr_*x*_Bi_2_Se_3_. J. Am. Chem. Soc. 137, 10512 (2015). 26262431 10.1021/jacs.5b06815

[b16] Dunsworth A. The de Haas-van Alphen effect in PdTe_2_. J. Low Temp. Phys. 19, 51 (1975).

[b17] Jan J. P. & Skriver H. L. Relativistic bandstructure and Fermi surface of PdTe_2_ by the LMTO method. J. Phys. F: Metal Phys. 7, 1719 (1977).

[b18] Ryan G. W. & Sheils W. L. Electronic states and surface structure of PdTe_2_ as probed by scanning tunneling microscopy and photoemission spectroscopy. Phys. Rev. B 61, 8526 (2000).

[b19] Liu Y. . Electronic structure of transition metal dichalcogenides PdTe_2_ and Cu_0.05_PdTe_2_ superconductors obtained by angle-resolved photoemission spectroscopy. Chin. Phys. B 24, 067401 (2015).

[b20] Furuseth S. . Redetermined crystal structures of NiTe_2_, PdTe_2_, PtS_2_, PtSe_2_, and PtTe_2_. Acta Chern. Scand. 19, 257 (1965).

[b21] Jaudet C. . de Haas-van Alphen oscillations in the underdoped high-temperature superconductor YBa_2_Cu_3_O_6.5_. Phys. Rev. Lett. 100, 187005 (2008). 18518412 10.1103/PhysRevLett.100.187005

[b22] Lawson B. J., Hor Y. & Li L. Quantum oscillations in the topological superconductor candidate Cu_0.25_Bi_2_Se_3_. Phys. Rev. Lett. 109, 226406 (2012). 23368142 10.1103/PhysRevLett.109.226406

[b23] Eun J. Theory of quantum oscillations in cuprate superconductors. *PhD Dissertation, University of California* (2012).

[b24] Lawson B. . Quantum oscillations in Cu_*x*_Bi_2_Se_3_ in high magnetic fields. Phys. Rev. B 90, 195141 (2014).

[b25] Forro L., Biljaković K., Cooper J. & Bechgaard K. Magnetoresistance of the organic superconductor bis-tetramethyltetraselenafulvalenium perchlorate [(TMTSF)_2_ClO_4_]: Kohler’s rule. Phys. Rev. B 29, 2839 (1984). 10.1103/physrevb.38.11177 9945992

[b26] Shekhar C. . Extremely large magnetoresistance and ultrahigh mobility in the topological Weyl semimetal candidate NbP. Nature Phys. 11, 645 (2015).

[b27] Liang T. . Ultrahigh mobility and giant magnetoresistance in the Dirac semimetal Cd_3_As_2_. Nature Mater. 14, 280 (2015). 25419815 10.1038/nmat4143

[b28] Kriener M., Segawa K., Ren Z., Sasaki S. & Ando Y. Bulk Superconducting Phase with a Full Energy Gap in the Doped Topological Insulator Cu_*x*_Bi_2_Se_3_. Phys. Rev. Lett. 106, 127004 (2011). 21517345 10.1103/PhysRevLett.106.127004

[b29] Wang Z. W. . Superconductivity in Tl_0.6_Bi_2_Se_3_ Derived from a Topological Insulator. Chem. Mater. 28, 779 (2016).

